# Examining the key determinants of the jordanian customer’s adoption of genetically modified food

**DOI:** 10.1016/j.heliyon.2023.e16920

**Published:** 2023-06-03

**Authors:** Ali Abdallah Alalwan, Saeid Abu-Romman, Ghazi Al-Weshah, Yogesh K. Dwivedi, Hanaa Albanna

**Affiliations:** a Department of Management and Marketing, College of Business and Economics, Qatar University, P.O. Box - 2713, Doha, Qatar; b Al-Balqa Applied University, Department of Biotechnology, Al-Salt 19117, Jordan; c Department of Marketing, Faculty of Business, Al-Balqa Applied University, Jordan; d Digital Futures for Sustainable Business & Society Research Group, School of Management, Swansea University, Bay Campus, Fabian Bay, Swansea, SA1 8EN, Wales, UK; e Department of Management, Symbiosis Institute of Business Management, Pune & Symbiosis International (Deemed University), Pune, Maharashtra, India; f Northumbria University London (QAHE) London, UK

**Keywords:** GMF, Jordan, Adoption, Customers, DOI

## Abstract

Genetically modified food (GMF) is one of the most debated issues in the food market. There has been considerable interest from both academic researchers and policy makers regarding the antecedents and consequences of the commercial adoption of GMF applications. Conceptually, GMF can be defined as “Genetically modified (hereafter GM) foods are produced from genetically modified seeds or ingredients derived from plants or animals whose DNA has been manipulated using genetic engineering methods” [1, p. 2861]. However, only a limited number of studies have tested the related issues of GMF products from a customer perspective. Thus, this project intends to discover and examine the main drivers and hindrances in predicting customers’ intention and buying decision behaviour in developing Arabian countries (i.e., Jordan). A diffusion of innovations (DOIs) model was selected as the theoretical basis for the current study project. A field survey study was conducted to collect the requested quantitative data from a convenience sample of Jordanian customers. Statistical results largely supported the role of relative advantage, compatibility, trialability, social approval, awareness, perceived risk and price value on the behavioural intention to adopt GMF products, which in turn significantly predicted actual adoption behaviour. The results of the current project will hopefully expand the current academic understanding of the main factors that predict Jordanian customers’ perception and adoption of GMF products.

## 1 Introduction

Increasingly, GMF represents a growing area of business and investment, and a huge amount of financial resources and land has been devoted to its development. For example, by the end of 2018, the total area of agricultural land allocated for the production of GMF crops was approximately 191.7 million hectares [2]. Furthermore, in the USA food market, the GMF industry comprises approximately 70% of the food sector, as reported by Aarlberg [3]. The worldwide expansion in investment in the GMF industry could result in added value and contributions that could be captured by adopting GMF applications in terms of cost reduction, food innovation, more effective and efficient agricultural production, sustainable supply processes, and competitive prices [4].

Similarly, GMF technology has been largely approved as a new mechanism and has been adopted worldwide to address issues related to the environment and agricultural development [1]. For example, the National Research Council [5] confirmed that GMF technology has contributed greatly to reducing the rate of environmental pollution resulting from the use of pesticides and chemicals. Due to its abundance of crops and its high quality, GMF technology has also empowered developing countries to maintain their plant cover from forests and their biological diversity [6]. Another contribution of GMF technology is related to its ability to produce agricultural crops with more efficient use of water as well as a more efficient way to address problems related to microbial contamination [7]. Accordingly, GMF has been among the main food items shopped and consumed on a daily basis [1].

As customers seem to be more aware and engaged with issues related to the preservation of the environment and moral and social responsibility, the success of GMF is neither an inevitable nor a guaranteed outcome but largely depends on customers’ knowledge and their willingness to accept GMF applications [i.e. 8]. Thus, researchers in marketing and consumer areas have argued the pros and cons of this emerging food technology, as reported by Kim et al. [9], Gaskell and Stares [10]. A positive indication is that, according to the National Research Council [5], people progressively perceive consuming GMF products as not risky and similar to other types of traditional and organic foods.

Another perspective has considered how the consumption of GMF foods comprises a degree of uncertainty and risk from the customer’s point of view in both developed and developing countries [11]. One of the main causes of customers’ reluctance towards GMF foods could be their lack of knowledge and mistaken beliefs [12]. This, in turn, might negatively impact customers’ attitudes and willingness to buy and consume GMF products, as argued by Wunderlich and Gatto [13]. Customers are also more likely to be unsure about the consequences of using GMF products, especially with regard to its environmental and health impact [1]. In fact, the reactions of customers and organisations to GMF products has been found to be different from culture to culture; while US customers increasingly adopt such new foods, a high level of resistance has been found among European customers [14; 15). According to Costa-Font and Gil [16] and Platania and Pivitera [17], organic foods made based on typical cultural styles and food habits are preferred by the majority of European customers (i.e., Italy). Thus, producers of GMF products are usually concerned about the future of their business due to negative social reactions and resistance to the adoption of GMF products [18]. In fact, the lack of transparency and customer knowledge about GMF products have heightened customers’ concerns and decreased their motivation towards GMF products [1, 15, 19, 20]).

These common issues related to customers’ reluctance and feeling that GMF products are insecure have recently been reported by many studies worldwide conducted in places including Russia [21], the USA [ [22,23]], Australia [24], and China [25]. For example, in their quantitative analyses Marques et al. [24] found Australian customers to have negative attitudes towards GMF products. According to a cross-cultural study recently conducted by Komoto et al. [26], neither Japanese nor French consumers support the consumption of GMF products. Based on the results of two studies undertaken in the USA by Gwira Baumblatt et al. [23] and Scott et al. [22], more than half of the sample participants were against the use of GMF products. In their study of the Chinese food market, Zhang et al. [25] found a lower adoption rate of GMF products (30%) among participants in a targeted sample.

It is also important to argue that the success of GMF products largely depends on a solid foundation of a full understanding regarding the aspects that shape the customer’s emotional, cognitive, and behavioural reaction [27]. This challenge is evident and impressive in the context of developing countries due to customers’ lack of knowledge and awareness of the features of GMF products or even their existence in retail stores. Such a problem is more critical for the producers of GMF products in developing countries due to the lack of understanding of customers’ behaviour and perceptions. This is because few studies have examined the related issues of customers’ behaviour and perception towards GMF products. In this instance, a quantitative survey study undertaken within the Iranian food market indicated that customers are more likely to adopt GMF products as long as they fully trust, have few concerns, and have positive attitudes towards such food items [28]. Furthermore, the dimensions related to customers’ beliefs towards new products and food (i.e., food technology neophobia) could play an important role in hindering customers’ willingness to accept GMF as a new type of food product. In line with this assumption, in a study conducted by Kim et al. [9], food neophobia was confirmed to significantly moderate the impact of attitudes and ecological concerns on customers’ intention to consume GMF products in the South Korean context.

In light of the above discussion, it could be concluded that the success of marketing and promoting GMF products within the Jordanian context is not easy or guaranteed but rather depends on an adequate understanding of the Jordanian customer’s mindset, behaviour, and fears related to such new food types. Such an understanding requires a deep analysis and scientific study of the main factors that could be considered by Jordanian customers in adopting or rejecting such new food types. However, a critical review of the main body of literature shows that quite a few studies have addressed the related issues of GMF products from the perspective of customers in the Middle East [i.e., 28] and the Arab world. Moreover, this area has not yet been examined and discovered in the Jordanian economy, and no study has been undertaken in this regard from the point of view of Jordanian customers.

From a theoretical perspective, the current study would add value to the current literature on GMF products by proposing and defining the most relevant factors that would shape the buying intention toward such a particular kind of products. For example, the current study has recognised a need to combine factors from similar literature in the field of GMF in general with other closely related literature in innovation adoption and marketing. This study has also noticed the absence of studies testing the related issues of GMF adoption in the Middle East in general and in Jordan in particular, this study was able to make a considerable contribution by focusing on customers in areas that have received little attention from prior studies. Further, based on a careful review of the main body of literature, there is a lack of GMF studies that have considered the impact of innovation features proposed by Rogers [29] and Tornatzky and Klein [30]. This, in turn, is considered as a gap to be covered in the current study. A closer look at the main body of GMF literature also leads us to notice that a number of external factors (perceived risk, awareness, price value) were not fully covered by prior literature, and therefore, this study concentrates on integrating them into the current study model. These factors were integrated with innovative features proposed by Rogers [29] and Tornatzky and Klein [30], which in turn helps the current study to propose a comprehensive model covering the most important aspects from the perspective of Jordanian customers.

From a practical perspective, the traders and producers of GMF products have struggled to discover the main drivers and hindrances of consumers’ attitudes, perceived risk and intention to buy such food products. This is especially the case in light of the absence of the agreement of scientific researchers and practitioners regarding the pros and cons of GMF products. This in turn has created a blurred picture and increased the degree of uncertainty for the consumer towards GMF products. Accordingly, there is a constant need to explore the main aspects that could shape customers’ perception and buying behaviour towards GMF products. However, only a limited number of studies have tested the related issues of GMF products from the customer perspective. Thus, this project intends to discover and examine the main drivers and hindrances predicting customers’ attitudes and buying decision behaviour in developing Arabian countries (i.e., Jordan).

Accordingly, the results of the current project will hopefully expand the current academic understanding towards the main factors predicting Jordanian customers’ perception and adoption of GMF products. Similarly, Jordanian organisations, which are engaged in producing and trading GMF products, will learn more how they could professionally design and manage their marketing activities to accelerate positive awareness among Jordanian customers towards GMF products. This, in turn, will positively reflect Jordanian customers’ intention and adoption of GMF products and hinder the neophobia from which Jordanian customers could suffer in dealing with and using GMF as new foods produced using new technologies.

In the light of the above-mentioned discussion, the research questions of the current study would be summarised as follow:

Q1: What are the main drivers that could positively impact the Jordanian customers’ intention to adopt GMF products?

Q2: What are the main hindrances that could negatively impact the Jordanian customers’ intention to adopt GMF products?

Q3: How would the Jordanian customers’ intention impact the actual adoption behaviour of GMF products?

## 2 Literature review

Genetically modified foods have been controversial issues receiving considerable attention from researchers in the food market area i.e., [9, 28, 31, 32]. The vast majority of these studies have argued the main factors that could determine customers’ attitudes, perceptions, and adoption of such new types of food [i.e. 1]. Researchers have proposed and validated various factors that could either hinder or drive customer adoption and attitudes towards GMF products.

Customers’ fears and perceived risk have been commonly cited as key factors hindering customers’ intention and adoption of GMF products. In their recent study, Delmond et al. [21] empirically approved the impact of perceived risk on customers’ decision to adopt GMF products in the Russian market. According to Royzman et al. [33], customers are more likely to formulate unfavourable attitudes towards GMF due to concerns about consuming such products and fears related to their impact on their health. Kim et al. [9] were also able to confirm the negative impact of ecological concerns on both customers’ attitudes and intention to purchase GMF products.

A lack of customer knowledge of and resistance to new food technologies were also among the key barriers hindering customers’ buying behaviour towards GMF products, as reported by Cobb and Macoubrie [34]. In line with this proposition, recent studies conducted by Boccia et al. [32] and Kim et al. [35] have articulated that the extent to which customers could adopt and formulate positive preferences towards consuming GMF products largely depends on the knowledge they possess. Therefore, these studies supported the impact of the education level on the level of knowledge towards GMF products and, accordingly, customer perception and intention.

Another part of the literature has focused on the light side of consumer reaction towards GMF products by either considering the main factors motivating customers to adopt such emerging food items or the benefits perceived by customers. Phillips and Hallman [36] noted that GMF products are largely perceived by customers to have richer ingredients and nutrients and are more reasonably priced than traditional types of food products. Similarly, Grunert et al. [37] discussed aspects of product quality in terms of sensible properties such as food shape and taste as key utilities and benefits that customers could capture from buying and consuming GMF products.

In their empirical study undertaken in the Italian food market, Pino et al. [1] found that Italian customers’ positive attitudes and intention to adopt GMF products largely depend on the extent to which producers of such products carry out their social responsibility properly, especially in terms of philanthropic and legal responsibilities. Similarly, both customer trust and customer perception of corporate social responsibility were also confirmed by Akbari et al. [28] to have a significant influence on Iranian customers’ tendency to buy GMF products.

The impact of cultural dimensions and values (i.e., egalitarian communitarian and hierarchical-individualistic worldviews) on customers’ willingness to adopt GMF products were argued by Kemper et al. [38]. Kemper et al. [38] observed that individuals in hierarchical-individualist cultures are more likely to support the evolution of food technology production and to prefer to adopt GMF products. On the other hand, customers in communitarian contexts were found to be less interested in consuming GMF products as they are willing to pay higher prices to buy products that are labelled as non-genetically modified foods [38].

Although these studies have contributed considerably to the current understanding of GMF products from the customer perspective, there are still a number of issues that call for further analyses and examination. For instance, examining the customer’s adoption of GMF products should be based on a solid theoretical foundation that is able to cover the most important factors that could hinder or contribute to the customer’s intention and adoption of GMF products. In this regard, it is important to indicate that the theory of planned behaviour (TPB) has been the most common theory used by researchers who have tested the adoption of GMF by customers [i.e., 28; 9; 8]. However, the TPB has been criticised for not covering important aspects that could predict human behaviour towards new innovations such as technologies, new products, and novel food types (i.e., GMF) [39]. For example, TPB has ignored the important impact of customers’ psychological personality and demographic characteristics [[39], [40], [41]]. Therefore, the TPB has constantly suffered from its inability to obtain an adequate level of predictive validity [39]. According to a meta-analysis study conducted by Armitage and Conner [39] who reviewed approximately 185 studies that formulated TPB, not more than 39% of the variance was accounted for by three TPB constructs (subjective norms, perceived behavioural control, and attitudes) in behavioural intention. Later, Akbari et al. [28] expanded the theoretical horizon of TPB by adding new aspects of regulatory focus theory. However, the results yielded by Akbari et al. [28] disapproved of the impact of one of the important dimensions (perceived behavioural control) on the customer’s intention. Cultural cognition theory was also considered by Kemper et al. [38], yet this theory considers only the cultural aspects while ignoring the main drivers or hindrances related to consumer behaviour regarding GMF products.

Importantly, to the best of the researcher’s knowledge, the related issues have never been discussed and tested in Arabian countries, especially in Jordan. Therefore, the first gap that this study seeks to fill is to find a solid theoretical foundation to propose the current project conceptual model. This model should provide a novel contribution by addressing the most important factors predicting customers’ intention and adoption of GMF products. Researchers are also keen to consider the particular nature of the Jordanian food markets, and therefore, the current project will be conducted in several successive stages, and it will consider different research techniques, as discussed in the proposed conceptual model and methodology section.

## 3 Conceptual model

Due to the novel nature of GMF products, there was a need to select a model addressing how customers could perceive and behave towards applications of food innovation. As Rogers’ [29] diffusion of innovations (DOIs) model was proposed to explain how people could accept or reject new innovations, it was selected as the theoretical basis for the current study project. In fact, DOI has been one of the most common models adopted by researchers in the context of an individual’s behaviour and new innovation [42]. Four main factors, relative advantage, lack of observability, trialability, and compatibility, were proposed by Rogers [29] as key drivers of the adoption behaviour of new innovation. Tornatzky and Klein [30] also considered other innovation features such as social approval. These aspects will be proposed along with four features proposed by Rogers [29].

**Fig. 1:**
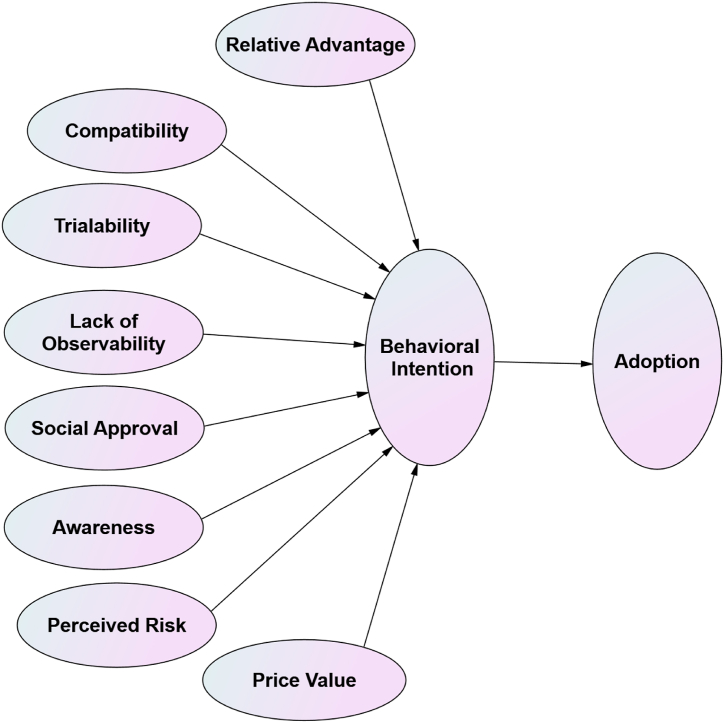
Conceptual model adapted from Rogers [29] and Tornatzky and Klein [30].

As the current project intends to provide a full picture of the main predictors of Jordanian customers’ intention and adoption of GMF, other factors that have been largely repeated and cited in the relevant literature will be included in the same conceptual model. Such factors include perceived risk [21], awareness [21] and price issues [4]. All of these factors were also included in the conceptual model (please see Fig. 1).

Considering the particular nature of the Jordanian food markets, researchers will also attempt to add other factors that could be important from the perspective of Jordanian customers. Practically, researchers organised a number of exploratory interviews with Jordanian customers as well as a number of specialists and experts engaged in producing and trading GMF products in the Jordanian food market. These interviews helped the researchers first approve the applicability of the innovation features adopted in the current model to the Jordanian context and to identify other external constructs that are highly weighted by Jordanian customers to adopt or reject GMF.

### 3.1 Relative advantage

In line with Rogers [ [29], p. 229], who asserted that “relative advantage is the degree to which an innovation is perceived as being better than the idea it supersedes”, customers’ intention and adoption of GMF products are largely shaped by the extent to which they perceive those products as being more useful and advantageous than traditional kinds of foods [43]. European countries appear willing to choose GM foods provided that there is a price advantage coupled with a consumer benefit (for example, spray-free GM fruits) [44]. For example, Knight et al. [44] noted that European customers were more enthusiastic about purchasing GMF products due to the relative advantage such as GM vegetables and fruits that are free of pesticides. Therefore, customers’ perceptions of benefits or relative advantages have been largely reported by the prior literature as a key determinant of customers’ intention and adoption of GMF products [i.e., [43,45]]. According to Weiss et al. [46], Chang et al. [47] and Siegrist et al. [48], the technology used in developing genetically modified food products contributes to their quality, taste and health benefits, which in turn causes GMF products to have a higher value than traditional types of food. Furthermore, Chen [43] argued that customers who view GMF products as comprising considerable benefits are more likely to override the associated risk of consuming such products and, accordingly, improve consumer acceptance of such products. Thus, this study proposes the following:

H1 Relative advantage will positively correlate with customers’ intention to adopt GMF products.

### 3.2 Compatibility

Rogers [ [29], p. 15] defined compatibility as the “degree to which an innovation is perceived as consistent with existing values, needs, and past experiences of potential adopters”. According to this definition, customers who perceive the consumption of GMF products to be compatible with their own thoughts, values, and lifestyles are more likely to adopt such products [49]. Furthermore, in the prior food marketing literature, the extent to which customers are familiar with the concept of GMF shapes their intention and purchasing behaviour towards such products [50]. Additionally, customers who perceive GMF products as being compatible with other types of food products that are typically purchased and consumed will be more motivated to adopt GMF products and new types of food products. In this respect, Siegrist et al. [49] argued that the compatibility of new food products with customers’ thoughts and attitudes towards health issues plays a key role in predicting customers’ intention to purchase. Furthermore, Menozzi et al. [51] provided empirical evidence approving the crucial role of compatibility in shaping customers’ decision to adopt new food products. Menozzi et al. [51] showed in detail that new food products that are perceived to be incompatible with local food culture have less chance of being adopted by customers. Thus, this study proposes the following:

H2 Compatibility will positively correlate with customers’ intention to adopt GMF products.

### 3.3 Trialability

According to Rogers [ [29], p. 258], trialability could be defined as the extent to which customers are able to experiment with new products on a limited basis without incurring any costs or commitment in the long term. Indeed, customers who are able to try new products would be more able to effectively evaluate the benefits of new products and, accordingly, be surer regarding their decision to accept or reject such products [52]. Flight et al. [53] also added that trialability also helps customers reduce the expected risks associated with such innovative products. The role of trialability usually appears clearly for potential adopters as they have more opportunity for magnitudes of change in their consumption patterns due to the use of new products [52]. Therefore, trialability has been commonly adopted by the prior literature for testing innovation diffusion and new product adoption [[53], [54], [55]]. All things considered, it could be suggested that customers who perceive GMF products to be triable and testable will be more certain regarding their decision behaviour towards such novel food products, and, accordingly, they will be more trusting of and motivated to buy such innovative foods in the short run. Accordingly, this study proposes the following:

H3 Trialability will positively correlate with customers’ intention to adopt GMF products.

### 3.4 Lack of observability

According to Rogers [29], observability can be articulated as the extent to which customers are able to physically and visibly experience the results of using new innovative products (GMF). Thus, observability is defined as the ability of customers to simply perceive and contact others regarding innovative products as well as the related outcomes of consuming such products [47]. Furthermore, innovative products that obtain a high level of observability are more likely to be adopted by customers, as reported by Makse and Volden [56] and Scott et al. [57]. However, how GMF has been improved and the related outcomes of its consumption are not easily observable i.e., [14, 16, 58]. Accordingly, one of the big challenges slowing the adoption of GMF products is the lack of observability. In other words, if customers perceive a lack of observability of GMF products, they will not be able to be familiar with such food products and, accordingly, be less motivated to adopt them [47]. Therefore, in line with the approach proposed by Chang et al. [47], this study tests the observability of GMF products. Therefore, lack of observability was defined in the current study as the extent to which outcomes of consuming GMF products are difficult for customers to see and touch. In the related area of GMF, several studies have shown that customers will not be motivated to buy GMF if they are not able to physically and visibly experience their benefits [58]. Thus, this study proposes the following:

H4 Lack of Observability will negatively correlate with customers’ intention to adopt GMF products.

### 3.5 Social approval

The social system plays a critical role in shaping how customers think, feel, and act towards products and brands, especially those with high levels of novelty and mystery such as GMF products [34, 47, 54]. Therefore, the social system will define which of such products will be accepted or rejected [59]. This may be based on the fact that customers return to their social system (i.e., reference groups) either to obtain the information they need regarding new products or to obtain social approval for their buying decision with regard to such products [47, 60]. In this respect, Chang et al. [47] argued that due to the lack of observability perceived in GMF products, customers have a highly intense need to consult their reference groups to know more about such products and to obtain more support for their buying decision towards GMF products. Consumers also have a greater need for the support and assurance of their surrounding community, especially in cases where the purchasing decision involves greater social, image and psychological risks. Parallel to the proposal of Chang et al. [47], it could be suggested that customers are more likely to adopt GMF products if they find that their social system largely approves and supports the buying and consuming of such food items. Thus, this study proposes the following:

H5Social approval will positively correlate with customers’ intention to adopt GMF products.

### 3.6 Awareness

The benefits involved in the use of GMF products will not be sufficient to encourage the consumer to purchase and consume such products if it is not associated with an adequate level of awareness and knowledge of such benefits and the reality of such products [15]. Therefore, customers’ awareness and knowledge have also been identified as key determinants of customers’ intention and adoption of GMF products [i.e., 43; 61; 62; 63]. In fact, the absence of customers’ awareness and knowledge regarding the main aspects of GMF products has been reported as a key inhibitor of customers’ adoption of such products [i.e., 16; 61; 43]. Conceptually, awareness could be defined as the extent to which customers know of the existence of GMF products and have adequate understanding and information about the nature, benefits, and even the associated risks of such products [43]. According to Chen [43], a lack of customer awareness and understanding creates negative attitudes and thus hinders customers’ willingness to adopt GMF products. Practically, customers’ knowledge not only accelerates their intention towards GMF products but also plays a key role in hindering the level of risk pertaining to such products as confirmed by Zhu et al. [62]. This could be attributed to the fact that an increase in the level of customers’ awareness of GMF products will reduce the level of uncertainty associated with consuming such products and accordingly cause customers to be more certain about their decision to adopt GMF products [i.e., 62]. Thus, this study proposes the following:

H5 Awareness will positively correlate with customers’ intention to adopt GMF products.

### 3.7 Perceived risk

Even though the expansion in producing and consuming GMF products has yielded many benefits to customers and contributed to customers’ eating habits, there are many barriers (i.e., perceived risk) that could hinder the success of using such technology as well as customers’ acceptance of such food products. Perceived risks have been commonly reported as key barriers to customers’ adoption of GMF products, as reported by Rodríguez-Entrena and Salazar-Ordóñez [63], Costa-Font and Gil [16] and Prati et al. [8]. Miles et al. [14] also added that the negative effects of GMF products are more unpredictable and usually long-term. Furthermore, the consumer’s lack of knowledge and inability to ascertain the results of consuming these foods were another cause for perceived risk, as indicated by Chen and Li [64] and Costa-Font et al. [16]. However, and according to Costa-Font et al. [16], perceived risk can be addressed in terms of customers’ perception, thoughts and beliefs that consuming GMF products will have negative effects on their health and psychological responses [62]. This, in turn, negatively reflects on how customers value such food products [65]. Accordingly, customers who perceive a high level of risk in consuming GMF are likely to be less motivated to adopt such food products, as empirically approved by Chen and Li [64]. Furthermore, the associated risks of GMF can be attributed to customers’ concerns regarding the moral and ethical issues of producing GMF products, which serve the aspirations of companies to obtain more profits rather than providing added value to the consumer [14]. Thus, this study proposes the following:

H6 Perceived risk will negatively correlate with customers’ intention to adopt GMF products.

### 3.8 Price value

In comparison with other kinds of natural and organic foods, producing GMF products is more cost-effective, and, accordingly, such products are more competitively priced [44, 66]. Therefore, price value has been widely reported as a key driver that accelerates customers’ intention and adoption of GMF products. Price value could be defined as the outcomes of the customer’s cognitive process of comparing the costs paid to obtain the required products and the expected benefits from these products. As long as customers perceive that using GMF products comprises more benefits and utilities than the cost paid, a higher price value will be perceived, and, accordingly, they will have a higher intention to adopt GMF products. The important role of price value could be because the vast majority of customers are of middle and lower income levels and are therefore more price sensitive. This, in turn, makes this large segment of customers more interested in GMF products due to their competitive prices. In fact, the role of price value in shaping customers’ intention and adoption of GMF products has rarely been examined in prior studies, as reported by Tuu and Olsen [67]. Accordingly, this study attempts to examine the critical role of price value in shaping Jordanian customers’ intention to adopt GMF products. Thus, this study proposes the following:

H7 Price value will positively correlate with customers’ intention to adopt GMF products.

### 3.9 Behavioural intention and GMO foods adoption

Compared to the rapid adaptation of GMO technologies in the market, consumer adoption of GMO products has been a topic of significant debate. Scholars have made significant efforts to examine market expectations and valuations of GMO foods. Hess et al. [68] reviewed 214 journal articles and government reports published between 1991 and 2012 and reported that prior studies have placed a massive focus on examining the impact of customers’ perceptions, purchase intentions and willingness to pay, consumer attitudes towards GMO foods, and actual purchase and adoption of GMO foods. Actual purchase behaviour is a key point for consumers when selecting and evaluating such products [69]. Ghosh [70] claimed that purchasing intention is an important instrument that is used to determine the purchase process. When consumers decide to buy the commodity in a specific shop, they will be directed by their intention. Therefore, purchase intention is a major predictor of customer behaviour. In other words, behavioural intention or purchase intention describes the ability and potential of customers to engage in actual purchasing behaviour towards GMF products. A favourable buying experience reinforces the consumer’s desire to buy again and, accordingly, sustains adoption behaviour towards GMF products. The more regular consumers obtain good experiences from a firm, the more likely they will be to do business with such products. Accordingly, this study proposes the following:

H8 The customer’s behavioural intention will influence the customer’s actual adoption of GMF products.

## 4 Research methodology

In line with the discussion in the conceptual model section, this project attempts to obtain an accurate picture of the adoption of GMF products from the perspective of Jordanian customers. Therefore, a field survey study was conducted to collect quantitative data from Jordanian customers in the period from September 2021 to the end of December 2021. Therefore, a questionnaire was selected as the main data collection instrument. A convenience sampling technique was also employed to reach the targeted Jordanian customers who were asked to complete the questionnaire. Due to the lack of an up-to-date and reliable list of Jordanian customers, the researchers opted to use convenience sampling in their study. This sampling technique was chosen because the community of Jordanian customers is large and dispersed over a wide geographical area. Convenience sampling is also a popular and commonly used sampling technique in organisational and consumer studies. Hair et al. [71] recommend a sample size between 200 and 400 as it is more accurate and suitable. A sample size above 400 can make the maximum likelihood estimation more sensitive, and certain fit indices such as chi-square may indicate a poor fit model when a larger sample size is used [[72], [73], [74]]. Accordingly, the sample size in the present study (249), was appropriate for further analysis using SEM.

The main constructs of the current study were measured based on the scale items derived from their original research sources. For example, the items of relative advantage were extracted from Miles [14] and Chang et al. [47]; the social approval items were derived from Chang et al. [47] and Huang (2018); the lack of observability items were derived from Chang et al. [47]; the compatibility items were derived from Moore and Benbasat [75]; the purchase intention items were taken from Zhu [76]; the trialability items were taken from Moore and Benbasat [75]; the price value items were derived from Venkatesh et al. [40]; the scale proposed by Wee et al. [77] was adopted to measure the adoption behaviour; the scale items of awareness were selected from Miles [14]; and, finally, the perceived risk items were taken from Zhu [76], and Lopez [78] (see Appendix).

Actually, these items have been selected as they are suitable to the measurement of the current study problem (adoption of GMF products). This has been later assured by experts who judged the validity of the questionnaire. All of these items have been used to measure the customers’ adoption behaviour in several sectors (food; technology adoption; digital marketing; adoption of new products). Statistically, these items have always been supported to adequately match the criteria related to validity and reliability over the prior studies. Furthermore, all of these items have been extracted as they are from highly cited papers published in reputable journals.

As this study mainly targets Jordanian customers whose native language is Arabic, all scale items were translated using the back translation method suggested by Brislin [79] to avoid the impact of cultural differences. The Arabic version of the questionnaire was also validated by a number of experts in the areas of marketing and GMF. The feedback provided by those experts largely supported the adequacy of the current scale items to measure the targeted constructs, and they ensured that the Arabic version was identical in meaning and content to the original English version. Furthermore, a pilot study with 30 participants was also conducted in the current research to determine the appropriateness of the time required to complete the questionnaire and the language used from the point of view of Jordanian customers. The average time taken by the pilot study participants was approximately 15 min, and most of those participants reported that the language used in the questionnaire was clear and did not require much effort to be understood. However, there were a few comments regarding the repetition of some construct items that were carefully revised; however, the decision was to keep them as they were proposed in their original sources. A five-point Likert scale ranging from strongly agree to strongly disagree was used to capture the participants’ responses on the construct items used in the current survey.

It is also important to indicate that the authors whose names are listed in this paper (see title page) certify that they have NO affiliations with or involvement in any organisation or entity with any financial interest or non-financial interest in the subject of genetically modified food discussed in the current manuscript. Furthermore, the data instrument of the current study was approved from Al-Balqa Applied University in Jordan. Yet, this study has only targeted customers aged above 18 and avoids collecting data from children or patients.

Informed consent was obtained from all participants in the study. Specifically, the researchers initially provided potential respondents with comprehensive information regarding the nature of the survey, its main objective, the key benefits, and potential risks involved. All of this information was presented on the cover page of the questionnaire. As stated in the cover page documentation, respondents were assured that their participation was voluntary and that they had the freedom to choose not to complete the questionnaire without facing any consequences. Additionally, respondents were encouraged to contact the researchers using the provided contact details (such as email addresses and phone numbers) if they had any questions or inquiries. Furthermore, confidentiality was given careful consideration in the study, with the researcher assuring participants that the collected data would be treated confidentially and used solely for scientific research purposes.

## 5 Results

### 5.1 Demographic characteristics of study sample

In this study, a number of demographic variables were considered in the survey (gender, age range, education level, income level). The percentage of females (59.2%) was higher than the percentage of males (40.8%). The highest percentage among age groups was for the age category 18–24 years at 41.6%, while the lowest percentage among age groups was 0.9% for those 60 years or more. The age group 25–30 years comprised 31.7%, the age group 31–40 years comprised 14.4%, the age group 41–50 years comprised 6.4%, and the age group 51–60 years comprised 5%. The highest percentage among qualifications was for the academic qualification (bachelor’s degree) (69.6%), while the lowest percentage among educational qualifications (others) was 0.9%. The other educational level of the respondents observed (11.1%) was a master’s degree followed by high school qualification (8.3%) and a diploma degree (6.6%). A very small percentage of respondents (3.3%) held PhD degrees. The highest percentage among qualifications was for the academic qualification (bachelor’s degree) (69.6%), while the lowest percentage among educational qualifications (others) was 0.9%. The other educational level of the observed respondents (11.1%) had acquired a master’s degree followed by high school qualification (8.3%) and a diploma degree (6.6%).

### 5.2 Descriptive statistics

A five-point Likert scale was adopted in the current study to measure the degree of consent of the sample respondents to the scale items. As shown in Table 1, the perceived risk items account for the largest average mean value of 3.72 with a Std. deviation value of 0.96, which reflects that the respondents in the current study perceive that consuming GMF products is not safe and pose a a high level of health risks. The participants in the current study were also found to have an adequate level of awareness and knowledge regarding the GMF products as the average mean of the awareness scale items was 3.30 with a Std. deviation value of 1.07. The social approval items accounted for an average mean value of 3.10 with a Std. deviation of 1.14. This, in turn, reflects that Jordanian customers are influenced by the social system around them in terms of related purchasing decisions of GMF products. GMF products are more likely to be perceived as complicated and not easily understood by the sample participants due to the average mean of complexity items, which was 3.25, and a Std. deviation of 0.95. On the other hand, the participants in the current study were more likely to be neutral in their attitudes towards the benefits of consuming GMF products as the average mean value of the relative advantage items was 2.91 with a Std. deviation of 1.01. The items of compatibility were also moderately rated by the respondents of the sample with an average mean of 2.83 and a standard deviation of 0.93. The items of the scale of value for money and trialability are rated neutrally by the respondents with average mean values of 2.92 and 2.88 respectively. The purchase intention items have an average mean of 2.92 and a standard deviation of 1.07. The lowest average mean (2.64) is for the adoption scale items, indicating a lower acceptance of GMF products in Jordan.

**Table 1:** Descriptive statistics (mean and std. Deviation).

Construct	Item	Mean	Std. Deviation
Awareness	AW1	3.46	1.013
	AW2	3.04	1.06
	AW3	2.91	1.07
	AW4	3.71	1.13
	AW5	3.37	1.10
	Average	3.30	1.07
Perceived Risk	RS1	3.97	.89
	RS2	3.72	.92
	RS3	3.74	.95
	RS4	3.77	.97
	RS5	3.58	1.05
	RS6	3.52	1.00
	Average	3.72	.96
Social Approval	SA1	2.95	1.16
	SA2	2.99	1.17
	SA3	3.35	1.09
	Average	3.10	1.14
Relative Advantage	RA1	3.00	1.00
	RA2	2.61	1.03
	RA3	2.99	.97
	RA4	3.04	1.06
	Average	2.91	1.01
Purchase Intention	PI1	2.91	1.049
	PI2	2.90	1.09
	PI3	2.93	1.07
	Average	2.916	1.07
Lack of Observability	LOC1	3.08	1.01
	LOC2	2.8710	.97
	LOC3	3.16	.91
	LOC4	3.29	1.03
	LOC5	3.25	.95
	Average	3.13	.97
Compatibility	CP1	3.01	.86
	CP2	2.83	.88
	CP3	2.79	.96
	CP4	2.68	.98
	Average	2.83	.92
Trialability	TR1	2.67	1.076
	TR2	2.88	1.04
	TR3	2.99	1.00
	TR4	2.95	1.06
	Average	2.87	1.04
Price Value	PV1	2.96	1.00
	PV2	2.91	1.00
	PV3	2.87	.961
	Average	2.92	.99
Adoption	Adoption1	2.90	.91
	Adoption2	2.62	1.12
	Adoption3	2.50	1.08
	Adoption4	2.47	1.03
	Adoption5	2.7	.99
	Average	2.64	1.02

### 5.3 Normality

To ensure that the data collected was normally distributed and avoid any issues of non-normality, the researchers examined the actual data distribution to determine if it was normally and symmetrically distributed. To test for univariate normality for each variable, the skewness-kurtosis approach was used, as recommended by Byrne [80], Hair et al. [81] and Kline [82]. The statistical values of skewness and kurtosis were tested using AMOS21, and the values indicated that the data set was within their respective levels, supporting the normality of univariate distribution. As seen in Table 2, all values of skewness were below their cut-off point of 3, and all values of kurtosis were not more than 8, in accordance with Kline [81] and West et al. [83].

**Table 2:** Assessment of normality.

Variable	skew	kurtosis
AW1	−.356	−.403
AW2	−.160	−.526
AW3	−.247	−.566
AW4	.030	−.774
AW5	−.338	−.885
RS1	−.425	−.271
RS2	−.487	−.100
RS3	−.531	−.236
RS4	−.484	−.162
RS5	−.583	.311
RS6	−.496	−.587
SA1	−.196	−.993
SA2	−.164	−1.058
SA2	−.167	−1.099
RA1	−.121	−.474
RA2	−.369	−.318
RA3	.348	−.271
RA4	−.248	−.209
TR1	−.232	−.472
TR2	−.107	−.718
TR3	.057	−.741
TR4	.043	−.881
PV1	−.222	−.221
PV2	−.170	−.435
PV3	−.125	−.439
CP1	.158	−.450
CP2	.091	−.522
CP3	−.358	−.608
CP4	−.182	−.232
LOC1	−.178	−.681
LOC2	−.001	−.645
LOC3	.041	−.329
LOC4	.087	−.338
LOC5	−.405	−.429
PI1	−.144	−.671
PI2	−.221	−.826
PI3	−.202	−.665
RS1	−.425	−.271
RS2	−.487	−.100
RS3	−.531	−.236
RS4	−.484	−.162
RS5	−.583	.311
RS6	−.496	−.587
Adoption1	.188	−.569
Adoption2	−.014	−.485
Adoption3	.304	−.626
Adoption4	.343	−.576
Adoption5	.255	−.700

### 5.4 Structural equation modeling analyses

A two-stage SEM approach was employed in the current study. This study utilised structural equation modeling (SEM), a statistical technique that is suitable for validating a conceptual model and testing research hypotheses. SEM is a set of statistical techniques that can examine the relationships between one or more independent variables and one or more dependent variables, whether continuous or discrete. SEM was chosen as the appropriate statistical tool because it can simultaneously investigate the relationships between observed variables (indicators) and non-observed variables (latent constructs), and can verify causal relationships between latent constructs using structural model analyses. In addition, SEM can evaluate the unidimensionality, reliability and validity of each construct individually, making it a useful tool for testing hypotheses and validating the proposed conceptual model. Confirmatory factor analysis (CFA) is often used to conduct such examinations within SEM. To perform the SEM for the current study analyses, the researchers utilised analysis of moment structures (AMOS21).

#### 5.4.1 Measurement model

##### 5.4.1.1 Model fitness

A number of fit indices were initially inspected to determine the extent to which the measurement model fit the observed data such as (CMIN/DF; GFI; AGFI; NFI; CFI; RMSEA). The initial results of the fit indices (i.e., CMIN/DF = 4.521; GFI = 0.793; AGFI = 0.712, RMSEA = 0.075; NFI = 0.841; CFI = 0.891) of the first version of the measurement model with all 52 scale items were not found to be within their threshold level (see Table 3). Thus, the measurement model was cleaned by dropping the most problematic items. A number of items were found to have a factor loading of less than 0.50 such as AW4, SA3, LOC4, TR4 and adoption1. The revised version of the measurement model without these problematic items was able to capture an adequate level of goodness of fit as all indices existed within their threshold level, i.e., CMIN/DF = 2.688; GFI = 0.931; AGFI = 0.882, RMSEA = 0.061; NFI = 0.961; CFI = 0.985.

**Table 3:** Results of measurement model.

Fit indices	Cut-off point	Initial measurement model	Modified measurement model
CMIN/DF	≤3.000	4.521	2.688
GFI	≥0.90	0.793	0.931
AGFI	≥0.80	0.712	0.882
NFI	≥0.90	0.841	0.961
CFI	≥0.90	0.891	0.985
RMSEA	≤0.08	0.075	0.061

##### 5.4.1.2 Construct reliability and validity

Three main criteria, average variance extracted (AVE), composite reliability (CR) and Cronbach’s alpha (α), were tested to ensure that all constructs with their unremoved items attained an acceptable level. The CR values for all latent constructs were noticed to be higher than 0.70 [i.e., 81] (see Table 4). Purchase intention was able to capture the highest value of CR (0.94), followed by actual adoption, with a value of 0.92, while awareness recorded the lowest CR value (0.80). Similarly, the Cronbach’s alpha (α) values for all constructs were found to be above 0.70 [84]. Purchase intention also accounted for the highest value of Cronbach’s alpha (α) (0.941), followed by actual adoption, with a Cronbach’s alpha (α) of 0.919. Awareness again had the lowest Cronbach’s alpha (α) value of 0.792; however, it was still above the threshold level of 0.70 suggested by Nunnally [84]. As seen in Table 4, all AVE values ranged between 0.507 (awareness) and 0.84 (purchase intention), which were higher than 0.50 and therefore acceptable according to Fornell and Larcker (1981) and Hair et al. [81].

**Table 4:** Construct reliability and validity.

	α	CR	AVE
PV	0.897	0.899	0.748
PI	0.941	0.943	0.845
RA	0.825	0.829	0.549
TR	0.819	0.820	0.603
LOC	0.805	0.808	0.517
CP	0.838	0.841	0.573
Adoption	0.919	0.922	0.703
RS	0.906	0.908	0.622
SA	0.824	0.827	0.706
AW	0.792	0.800	0.507

As shown in Table 5, all unremoved scale items were found to have standardised regression weight (factor loading) values not less than 0.50, as suggested by Hair et al. [81]. The purchase intention items, PI2, PI2 and PI3, have the highest factor loading values ranging between 0.897 and 0.938, while the lowest factor loading values were found for the scale items of awareness, which ranged between 0.551 (AW5) and 0.852 (AW3). Furthermore, all items significantly loaded on their latent constructs with *P* values less than 0.001 and C.R. values higher than 1.96 [81]. The constructs also matched the criteria of discriminant validity as the squared root of AVE for each latent construct was higher than the intercorrelation values with other corresponding constructs (see Table 6).

**Table 5:** Standardised regression weights (factor loading).

			Estimate			
AW1	<---	AW	.613	.142	7.024	***
AW2	<---	AW	.789	.148	9.196	***
AW3	<---	AW	.852	.161	9.103	***
AW5	<---	AW	.551	.135	7.254	***
RS1	<---	RS	.724	.058	17.241	***
RS2	<---	RS	.736	.092	11.456	***
RS3	<---	RS	.849	.096	12.930	***
RS4	<---	RS	.848	.098	13.079	***
RS5	<---	RS	.849	.109	12.651	***
RS6	<---	RS	.711	.104	10.618	***
SA1	<---	SA	.849	.058	17.241	***
SA2	<---	SA	.831	.061	16.147	***
PI1	<---	PI	.938	.051	19.607	***
PI2	<---	PI	.897	.042	23.812	***
PI3	<---	PI	.923	.038	26.948	***
RA1	<---	RA	.777	.101	11.126	***
RA2	<---	RA	.724	.104	10.346	***
RA3	<---	RA	.717	.101	9.900	***
RA4	<---	RA	.744	.103	10.968	***
LOC1	<---	LOC	.808	.143	9.304	***
LOC2	<---	LOC	.802	.139	9.161	***
LOC3	<---	LOC	.600	.112	8.008	***
LOC5	<---	LOC	.641	.103	9.708	***
CP1	<---	CP	.630	.195	5.128	***
CP2	<---	CP	.706	.122	9.466	***
CP3	<---	CP	.826	.141	10.465	***
CP4	<---	CP	.845	.148	10.415	***
PV1	<---	PV	.828	.058	17.241	***
PV2	<---	PV	.902	.063	17.169	***
PV3	<---	PV	.863	.062	16.049	***
TR1	<---	TR	.806	.095	10.526	***
TR2	<---	TR	.795	.081	11.801	***
TR3	<---	TR	.726	.077	10.851	***
adoption2	<---	Adoption	.881	.057	17.543	***
adoption3	<---	Adoption	.875	.048	19.686	***
adoption4	<---	Adoption	.845	.051	17.294	***
adoption5	<---	Adoption	.786	.051	15.306	***

**Table 6:** Discriminant validity.

	PV	PI	RA	TR	LOC	CP	Adoption	RS	SA	AW
PV	**0.865**									
PI	0.639	**0.919**								
RA	0.638	0.654	**0.741**							
TR	0.551	0.479	0.509	**0.776**						
LOC	0.282	0.232	0.326	0.369	**0.719**					
CP	0.659	0.708	0.672	0.542	0.404	**0.757**				
Adoption	0.662	0.610	0.623	0.623	0.309	0.660	**0.838**			
RS	−0.334	−0.557	−0.492	−0.189	−0.070	−0.381	−0.283	**0.789**		
SA	0.663	0.723	0.692	0.488	0.247	0.719	0.651	−0.660	**0.840**	
AW	0.258	0.251	0.204	0.257	0.122	0.399	0.247	0.133	0.109	**0.712**

#### 5.4.2 Multicollinearity test

The table displays that the Variance Inflation Factor (VIF) values, ranging from 1.874 to 2.974, were significantly lower than the recommended cut-off value of 10 by Brace et al. [85] and Diamantopoulos and Winklhofer [86], and Irani et al. [87]. This provides clear evidence that there is no need to be concerned about multicollinearity for the three samples in the current study.

#### 5.4.3 Common method bias

In order to address the related issues of common method bias, we have applied Harman’s single-factor test with 10 latent constructs (PV; PI; RA; TR; LOC; CP; Adoption; RS; SA; and AW) and 42 measurement questions [88,89]. As shown in Table 7, all of the measurement items were loaded into the exploratory factor analysis and tested using unrotated factor solution. Table 7 clearly shows that a single factor has not emerged and the first factor only reports 31.636% of variance. As this value (31.636) is not higher than 50% as highly recommended by Podsakoff et al. [89], there is no concern regarding the common method bias in the current study data.

**Table 7:** Common method bias test.

Total Variance Explained						
Component	Initial Eigenvalues			Extraction Sums of Squared Loadings		
	Total	% of Variance	Cumulative %	Total	% of Variance	Cumulative %
1	13.287	31.636	31.636	13.287	31.636	31.636
2	4.540	10.809	42.445			
3	2.779	6.617	49.061			
4	2.220	5.285	54.346			
5	2.013	4.792	59.138			
6	1.710	4.072	63.210			
7	1.294	3.082	66.292			
8	1.206	2.871	69.163			
9	1.099	2.617	71.780			
10	.984	2.342	74.122			
11	.899	2.140	76.263			
12	.869	2.069	78.332			
13	.776	1.848	80.180			
14	.713	1.698	81.879			
15	.660	1.572	83.451			
16	.576	1.372	84.823			
17	.537	1.278	86.102			
18	.508	1.210	87.312			
19	.479	1.140	88.452			
20	.436	1.039	89.491			
21	.406	.966	90.457			
22	.395	.939	91.396			
23	.367	.873	92.270			
24	.324	.773	93.042			
25	.296	.705	93.747			
26	.272	.647	94.393			
27	.246	.586	94.980			
28	.221	.525	95.505			
29	.216	.514	96.019			
30	.203	.483	96.502			
31	.190	.453	96.955			
32	.187	.444	97.399			
33	.168	.399	97.798			
34	.156	.371	98.169			
35	.146	.347	98.516			
36	.123	.292	98.808			
37	.118	.281	99.089			
38	.099	.236	99.325			
39	.089	.211	99.536			
40	.078	.186	99.722			
41	.071	.169	99.890			
42	.046	.110	100.000			
Extraction Method: Principal Component Analysis.						

An extra analysis was conducted to investigate whether there was a common method bias present in the dataset. This was done using the Common Latent Factor [89]. By using AMOS 22, it was recognised that the differences between standardised coefficients without a common latent factor and those with a common latent factor were less than 0.2. Consequently, it can be concluded that the presence of common method bias is not a significant concern in our dataset.

#### 5.4.4 Structural model analyses

In the second stage of SEM, the structural model, conceptual model goodness of fit, its predictive validity, and the nine research hypotheses were subjected to further analyses. The structural model was able to adequately fit the observed data as all fit indices were found to be within their suggested level; as such, CMIN/DF = 2.754; GFI = 0.918; AGFI = 0.861, RMSEA = 0.064; NFI = 0.952; CFI = 0.961. Furthermore, seven constructs accounted for approximately 0.71 of the variance in purchase intention, which also predicted approximately 0.45 of the variance in the actual adoption of GMF products. This, in turn, supported the predictive validity of the current study model. According to the path coefficient analyses, except complexity (γ = −0.091, p < 0.141), all predictors of purchase intention were supported in having a significant impact. Noticeably, compatibility (γ = 0.449, p < 0.000) was approved to have the most significant impact on purchase intention, followed by social approval (γ = 0.412, p < 0.000) (see Table 8). Relative advantage (γ = 0.223, p < 0.004), price value (γ = 0.1493, p < 0.005), awareness (γ = 0.133, p < 0.034) and trialability (γ = 0.124, p < 0.048) were also confirmed to have a significant influence on purchase intention. A strong and negative impact was approved for perceived risk (γ = −0.189, p < 0.003) on purchase intention (see Table 8). As expected, purchase intention largely predicts the actual adoption of GMF with a path coefficient value of 0.65 and a p value of 0.000 (see Table 8).

**Table 8:** Hypothesis testing.

Hypothesised path			Estimate	S.E.	C.R.	P	VIF
PI	<---	AW	.133	.063	2.121	.034	1.954
PI	<---	RS	−.189	.064	−2.970	.003	2.014
PI	<---	SA	.412	.067	6.168	***	2.637
PI	<---	RA	.223	.078	2.881	.004	2.146
PI	<---	LOC	−.091	.062	−1.474	.141	1.874
PI	<---	CP	.449	.103	4.350	***	2.974
PI	<---	PV	.149	.053	2.829	.005	2.654
PI	<---	TR	.124	.048	2.583	.048	2.159
Adoption	<---	PI	.650	.060	10.827	***	2.951

## 6 Discussion

This study has been conducted with the intention of providing further understanding regarding the extent to which Jordanian customers adopt and accept the purchase and consumption of GMF products. Furthermore, this study recognises the need to discover and test the key factors that could positively or negatively predict Jordanian customers’ intention and adoption of GMF products. As GMF products are more innovative and unique in comparison with traditional types of food, a number of innovative features derived from Rogers [29], and Tornatzky and Klein [30] were considered in the current study model as key determinants of the behavioural intention to adopt GMF products. Other factors (price value, social approval, awareness and perceived risk) were also integrated to expand the theoretical horizon of the current study model. The empirical part of the current study largely supported what has been proposed in the current study model. For example, the results of SEM largely supported the model goodness of fit and its predictive validity, as a large portion of variance was accounted for in both behavioural intention (0.70) and actual adoption behaviour (0.45).

According to path coefficient analyses, most of the research hypotheses were supported except for the impact of LOC on behavioural intention. Compatibility was found to have the strongest impact on behavioural intention. This means that Jordanian customers are more likely to be interested in adopting GMF products if they perceive such foods to be consistent with their values, thought, lifestyle and other kinds of food they are used to consuming. Such results of compatibility are in line with other studies that have supported the role of compatibility [i.e. 49, 35, 50]. A relative advantage was another innovative feature that obtained considerable attention from the current study sample in shaping their intention towards GMF products. In other words, customers who perceive GMF products as more valuable and beneficial in comparison with other kinds of traditional food will be more motivated to adopt such products. In fact, the innovative methods and techniques applied in producing GMF products contribute considerably in terms of quality, taste and health benefits that customers can obtain from consuming such products and, accordingly, enriching the perceived value in such innovative food products. Other studies, such as Chen [43] and Chang et al. [47], have achieved the same results of relative advantage yielded in the current study.

The path coefficient results also confirmed the significant role of trialability in predicting Jordanian customers’ intention to adopt GMF products. As argued in the conceptual model section, customers have more opportunities to experience the value and benefits of GMF products if they can try them without psychological, physical or financial consequences. Having the opportunity to experiment with GMF products also helps customers eliminate their perceived fears associated with these products. These results are parallel to those of other studies that have confirmed the important role of trialability in predicting consumer behaviour towards new innovations such as GMF products i.e., [5, 52, 55]. As expected, awareness has been supported by the current study results, which means that the opportunities to adopt GMF products increase by increasing customers’ awareness of such products. Such results can be attributed to the fact that there is a growing awareness of the importance and safety of GMF products and to the increased discussion and dialogue on all issues related to GMF products. In the prior literature on GMF, several studies have reached the same results of awareness as attained in the current study [i.e., [43,[61], [62], [63]]].

As found in the structural model results, the price value of GMF products presents an important factor considered by the current study sample in shaping their decision to adopt such products. By returning to the demographic characteristics of the current study sample, it can be noted that the vast majority of participants (0.65) had income levels of less than 600 JOD. This means that most of the sample participants were either middle- or lower-income customers, and, accordingly, they were more likely to pay particular attention to price issues. Furthermore, the cost of producing GMF products is very low compared with other kinds of organic and natural products, which makes their prices very competitive from the customer perspective [44].

According to the structural model results, the participants in the current study seemed to be concerned with the information, recommendations and opinions of the people surrounding them (i.e., friends, colleagues and family) regarding GMF products. Such results can be attributed to the fact that GMF products are more innovative and shrouded in much mystery, and, therefore, customers usually need their social system support and information to consume or avoid GMF products. Another justification of the significant role of social approval in the current study can be attributed to the nature of Jordanian culture, which is more of a collective culture in which social relations and social systems have great importance in determining the opinions and behaviour of individuals (Hofstede, 2001). Such results are not far from those of other studies supporting the role of social approval i.e. [34, 47, 54, 59].

Perceived risk was proposed as a negative factor hindering the behavioural intention to adopt GMF products. This proposition was statistically supported, as reported in the structural model analyses. In other words, customers are more likely to avoid buying and purchasing GMF products if they perceive such products as not safe and as having the potential to cause negative results and risks. Such expected risks can be attributed to the common and international argument regarding the negative side effects of GMF, especially in terms of genetic and animal welfare, thereby creating more doubts and negative reactions on the consumer side [8,62]. For example, Miles et al. [14] debated that GMF products would be a source of damage not only for human health and life but also the natural environment and future generations. Such results are similar to those reached by other studies in the related area of GMF products such as Rodríguez-Entrena and Salazar-Ordóñez [63], Costa-Font and Gil [16] and Prati et al. [8].

### 6.1 Theoretical contribution

By examining the most relevant factors affecting Jordanian consumers’ intention to purchase and adopt GMO products, the present study has made a major contribution by expanding the current understanding of GMF products. In terms of the value of clarifying how consumers formulate their intentions towards genetically modified foods, this study was initially able to make a fundamental contribution by combining similar literature in the field of GMF in general with other closely related literature in innovation adoption and marketing. More specifically, the present study reviewed the related studies in topics identifying key dimensions considered in GMF product investigations, and, accordingly, the study offered more knowledge and understanding of the key factors that should be taken into consideration when researching the intention of consumers to buy and consume GMF products. Therefore, the study's focus on Jordanian customers provides insights into the context-specific factors that influence consumer intention towards GMF products. Jordan is a unique context with its own cultural, social and economic characteristics that could affect consumer behaviour towards GMF products. The study's findings can help policymakers and marketers understand these context-specific factors and design effective strategies to promote the adoption of GMF products in Jordan.

In the absence of studies testing the related issues of GMF adoption in the Middle East in general and in Jordan in particular, this study was able to make a considerable contribution by focusing on customers in areas that have received little attention from prior studies. Therefore, the study contributes to the broader literature on consumer behaviour and innovation adoption by synthesising related literature and offering a comprehensive framework. The study's approach of combining literature on GMF products with related literature on innovation adoption and marketing could inspire other researchers to take a similar approach in their investigations. This approach could help to identify commonalities across different contexts and industries and promote cross-disciplinary research that could enhance our understanding of innovation adoption and consumer behaviour.

Furthermore, based on a careful review of the main body of literature, a number of external factors (perceived risk, awareness, price value) were integrated into the current study model. These factors were integrated with innovation features proposed by Rogers [29] and Tornatzky and Klein [30]. Thus, the study's comprehensive model covers various aspects that influence consumer intention towards GMF products. By integrating external factors, such as perceived risk, awareness and price value, into the innovation adoption model proposed by Rogers [29] and Tornatzky and Klein [30], the study offers a more complete understanding of the factors that influence consumer intention towards GMF products. This model could be used as a guide for future research and could also be applied by policymakers and marketers to design effective strategies to promote the adoption of GMF products.

### 6.2 Policy implications

The adoption of GMF products is not something to be taken for granted but rather requires effort on the part of marketers to persuade consumers to adopt GMF products as a suitable alternative food. This requires building a solid base of knowledge and understanding of the key reasons that cause consumers to either accept or reject GMF products. Thus, the results of the current study can help Jordanian organisations that are engaged in producing and trading GMF products to better understand how they could professionally design and manage their marketing activities to accelerate positive perception and adoption among Jordanian customers towards GMF products. For example, the significant impact of relative advantage provides clues for the marketers and producers of GMF products to give more attention in their promotional campaign to the key benefits and values of consuming such products. In this respect, the focus should not only be restricted to individual customers’ benefits but also its benefits to humanity in the field of food security and its impact on environmental suitability. Similarly, the role of compatibility requires more marketing effort convincing customers that GMF products are not different from the other kinds of traditional foods that customers are used to buying and eating as well as their eating lifestyle. As a new technology, customers are more likely to have more questions and inquiries regarding the nature, aspects and side effects of GMF products. Therefore, customer awareness, as defined in the current study, plays a key role in shaping customers’ intention and adoption towards GMF products. This could require GMF producers and marketers to create comprehensive content and information regarding all aspects related to GMF as technology and products. Such content should be customised in how it answers consumers’ questions and should create positive awareness towards these products. Furthermore, marketers can benefit from the revolution in the field of the internet and social media in delivering such content to the largest number of consumers in an appropriate and convincing manner.

Social approval impact means that marketing efforts should work on two levels: individual and societal. As the nature of Jordanian culture seems to be more collective, individuals’ decisions and behaviours are more influenced and shaped by the social system (family, friends and reference groups). This, in turn, requires more effort to create a positive culture and awareness in Jordan supporting the consuming and buying of GMF products. For instance, promotional campaigns should emphasise collective awareness and education about GMF products as healthy and safe foods. Perceived risk is found to be a negative factor hindering the customer’s willingness to adopt GMF products. Therefore, there is a need to initially understand which kinds of risk customers expect from consuming GMF products as well as the perceived main causes behind such risks. Addressing customers’ concerns and the perceived risk associated with GMF products would also require more efforts assuring consumers that these foods are safe and do not pose any health damage, whether in the short or long term. Due to the substantial influence of purchasing intention on the actual adoption of GMF along with the statistical findings (mean and standard deviation), it is apparent that the majority of respondents in the current study have a medium desire to adopt GMF products, and, accordingly, they may be deemed to be of valuable potential and interested adopters. Thus, transforming them into real consumers of GMF products is not expected to be costly and complicated. Communication is one of the main ways to reassure potential customers that GMF products are more useful than organic foods. In this respect, new advertising methods highlighting the benefits of GMF products compared to organic foods can be implemented using different media tools such as news media, social media, direct marketing or face-to-face communication.

## 7 Limitations and future research directions

Although this study has made a number of contributions and added to the current understanding regarding the related issues of GMF products, there are a number of limitations that could be considered by future studies. First, due to COVID-19 and its related restrictions, it was very difficult to personally contact the targeted respondents and reach them in their own homes. Accordingly, researchers collected the required data using an online questionnaire allocated to a convenience sample of Jordanian customers. This, in turn, negatively reflects the generalisability of the current results. Therefore, future studies should address issues of sampling bias and generalisability by using a more accurate sampling technique representing all components of the Jordanian community. Second, this study was cross-sectional, and both independent and dependent factors were tested at the same time. However, some aspects related to adoption behaviour could change over time. Accordingly, future studies will find it useful to determine how the current model factors behave differently over time by conducting longitudinal studies. Third, the main context of the empirical part of the current study was Jordan, which could hinder the applicability of the current study’s results to other countries, either in the Arab world or worldwide. Future studies are highly recommended to conduct cross-cultural studies to determine the impact of cultural differences on consumer behaviour towards GMF products. Finally, this study did not examine the moderating impact of gender, age, educational level or income level. Therefore, validating the moderation impact of these factors in future studies would provide an accurate picture of the differences between customer segments and accordingly help organisations identify suitable marketing strategies for each segment.

## Author contribution statement

Ali Alalwan: Conceived and designed the experiments; Performed the experiments; Wrote the paper.

Saeid Abu-Romman: Performed the experiments; Wrote the paper.

Ghazi Al-Weshah: Analyzed and interpreted the data; Wrote the paper.

Yogesh K Dwivedi; Hanaa Albanna: Contributed reagents, materials, analysis tools or data; Wrote the paper.

## Data availability statement

Data included in article/supp. material/referenced in article.

## Declaration of competing interest

The authors declare that they have no known competing financial interests or personal relationships that could have appeared to influence the work reported in this paper.

## Figures and Tables

**Table:** 

Construct	Items #	Item	Sources
**Relative advantage**	RA1	There would be benefits to consumers from genetic modification of food in the future.	Miles [14] and Chang et al. [47]
	RA2	GMO foods have more value than the items they are replacing.	
	RA3	GMO foods have the advantage of helping the body to absorb nutrition more easily.	
	RA4	Applying gene technology to produce food products will prove to be beneficial to the environment, myself and other people that are important to me.	
**Compatibility**	CP1	GM food is compatible with all other types of food I eat.	Benbasat [1991)
	CP2	GM food fits well with the type of food products that I always eat.	
	CP3	Eating GM food fits well with my lifestyle.	
	CP4	GM food is consistent with my beliefs and values.	
**Trialability**	TR1	I know grocery stores and retail which provides/offers GM food.	Moore and Benbasat [75]
	TR2	GM food was easily available to get familiar with.	
	TR3	I did not have to expend much effort in trying the GM food.	
	TR4	I have adequate opportunities to try the GM food.	
**Lack of observability (Complexity)**	LOC1	I would have difficulty telling others about the results of eating a GM food.	Chang et al. [47]
	LOC2	I believe I could not communicate to others the consequences of eating a GM food.	
	LOC3	I believe I could not communicate to others the consequences of eating a GM food.	
	LOC4	The results of eating a GM food would not be apparent to me.	
	LOC5	I would have difficulty explaining why eating A GM food may or may not be beneficial	
**Social approval**	SA1	If people around me suggest that I should try GMO foods, I will do it.	Chang et al. [47] and Huang (2018)
	SA2	If people around me think GMO foods are good and recommend them to me, I will try them.	
	SA3	My purchase or avoidance behaviour of GMO foods is influenced by my family and friends.	
**Awareness**	AW1	I have heard of genetically-modified foods.	Miles [14]
	AW2	I am personally very knowledgeable about the use of gene technology in food production.	Zhu [76] Miles [14]
	AW3	I need to know more information related to GMO food.	
	AW4	I am willing to know more information related to GMO food.	
	AW5	I know the difference between “genetically modified organisms” and “conventionally modified organisms".	
**Perceived risk**	RS1	There are harmful health effects on humans from consuming genetically-modified food.	
	RS2	Applying gene technology to produce food products involves considerable risk to the environment, myself and other people that are important to me.	Zhu [76] and Lopez [78]
	RS3	Applying gene technology to produce food products will prove disadvantageous to the environment, myself and other people that are important to me.	
	RS4	Eating GMO food will cause harm to my health as well as my family.	
	RS5	The production and consumption of GMO products is threatening to human nature.	
**Price value**	PV1	GM food is reasonably priced.	Venkatesh et al. [40]
	PV2	GM food is good value for money.	
	PV3	At the current price, GM food provides good value.	
	PV4	GM food is inexpensive.	
**Purchase intention**	PI1	If GM foods were available in the market, I would definitely buy them.	Zhu [76]
	PI2	If GM foods were available in the market, then I will plan to buy them.	
	PI3	If GM foods were available in the market, then I will try to buy them.	
**Adoption**	Adoption1	I often buy GM food products.	Wee et al. [77]
	Adoption2	I often buy GM food products on a regular basis.	
	Adoption3	I often buy GM food products because they are more environmentally friendly.	
	Adoption4	I often buy GM food products that are safe to consume.	
	Adoption5	I often buy GM food products for my health.	

## Appendix

Questionnaire.
